# Adipose-Derived Mesenchymal Cells for Bone Regereneration: State of the Art

**DOI:** 10.1155/2013/416391

**Published:** 2013-11-07

**Authors:** Marta Barba, Claudia Cicione, Camilla Bernardini, Fabrizio Michetti, Wanda Lattanzi

**Affiliations:** ^1^Institute of Anatomy and Cell Biology, Università Cattolica del Sacro Cuore, Largo F. Vito 1, 00168 Rome, Italy; ^2^Latium Musculoskeletal Tissue Bank, Largo F. Vito 1, 00168 Rome, Italy

## Abstract

Adipose tissue represents a hot topic in regenerative medicine because of the tissue source abundance, the relatively easy retrieval, and the inherent biological properties of mesenchymal stem cells residing in its stroma. Adipose-derived mesenchymal stem cells (ASCs) are indeed multipotent somatic stem cells exhibiting growth kinetics and plasticity, proved to induce efficient tissue regeneration in several biomedical applications. A defined consensus for their isolation, classification, and characterization has been very recently achieved. In particular, bone tissue reconstruction and regeneration based on ASCs has emerged as a promising approach to restore structure and function of bone compromised by injury or disease. ASCs have been used in combination with osteoinductive biomaterial and/or osteogenic molecules, in either static or dynamic culture systems, to improve bone regeneration in several animal models. To date, few clinical trials on ASC-based bone reconstruction have been concluded and proved effective. The aim of this review is to dissect the state of the art on ASC use in bone regenerative applications in the attempt to provide a comprehensive coverage of the topics, from the basic laboratory to recent clinical applications.

## 1. Introduction

Multipotent mesenchymal stem cells (MSCs) are nonhematopoietic cells of mesodermal derivation residing in several postnatal organs and connective tissues. They were first described in the early 1960s, as an adherent, fibroblastoid cell population with inherent osteogenic properties [1]. Since then, an overwhelming number of studies have demonstrated that MSCs are endowed with a higher plasticity, being able to differentiate into cells of mesenchymal lineages, such as adipogenic, osteogenic, and chondrogenic [2]. MSCs are also capable of transdifferentiation towards epithelial cells, such as alveolar epithelial cells [3], hepatocytes [4–7], epithelial cells from the gastrointestinal tract [8, 9], and kidney cells [10]. The question of possible neural transdifferentiation of MSCs is still debated and controversial [11–13]. Nonetheless, converging evidence has indicated the capability of MSCs to pursue a functionally and morphologically actual glial fate [14–17]. The common origin of both mesenchymal cells and neural cells from the neural crest, in the vertebrate embryo, may in part explain the high degree of plasticity of MSCs [18].

Bone Marrow (BM) was originally considered the reference source for MSCs isolation; to date they have been isolated from a multitude of adult tissues, including muscle, adipose tissue, connective tissue, trabecular bone, synovial fluid [19], and perinatal tissues, such as umbilical cord, amniotic fluid, and placenta [20–24]. In particular, the ubiquity, the ease of retrieval and the minimally invasive procedure required for harvesting the adipose tissue (AT), make it an ideal source for high yield MSCs isolation. Moreover, adipose tissue-derived MSCs (ASCs) can be maintained longer in culture and possess a higher proliferation capacity compared to BM-derived MSCs. Indeed ASCs and BM-MSCs exhibit virtually identical transcription profiles for genes related to the stem cell phenotype, supporting the concept of a common origin of the mesenchymal lineage from a wide variety of tissues [2, 25].

## 2. Fat as a Source of ASCs

Adipose tissue is a highly complex tissue comprising mature adipocytes (>90%) and a stromal vascular fraction (SVF), which includes preadipocytes, fibroblasts, vascular smooth muscle cells, endothelial cells, resident monocytes/macrophages, lymphocytes, and ASCs [26–28]. The density of the AT stem cell reservoir varies as a function of location, type, and species. Within the white fat, a highest number of ASCs reside in subcutaneous depots compared to visceral fat, with the highest concentrations occurring in the arm region and the greatest plasticity described in cells isolated from inguinal AT [29]. Studies in the canine model showed that the proliferative capacity of ASCs appears to inversely correlate with donor age, while stemness, self-renewal, and multipotency are progressively lost with culture passages [30, 31]. Moreover, significant differences in molecular profiles and immunophenotype have been described in subcutaneous and visceral fat-derived ASCs [31, 32]. The significant sexual dimorphism of adipose tissue distribution and function reflect gender- and hormone-related differences in cellular composition and molecular profiles, which should be taken in due account [33, 34]. Finally, ASCs have been described also in brown fat depots and are able to easily undergo skeletal myogenic differentiation [35, 36]. 

## 3. Isolation and *Ex Vivo* Expansion of ASCs

Human ASCs can be isolated from adipose tissue collected tissue through liposuction or during reconstructive surgery through resection of tissue fragments. Current methods used for isolating ASCs rely on collagenase digestion followed by centrifuge separation of the SVFs from primary adipocytes. ASCs are selected *in vitro* based on their plastic adherence properties and display typical spindle-shaped fibroblastoid morphology. They can be extensively subcultivated in monolayer culture on standard tissue culture plastics with a basal medium containing 10% of fetal bovine serum [2, 4, 37].

Once a primary culture is established, ASCs are easily and rapidly expanded *ex vivo* [2, 38]. The average frequency of ASCs in processed lipoaspirate is 2% of nucleated cells and the yield of ASCs is approximately 5,000 fibroblast colony-forming units (CFU-F) per gram of adipose tissue, compared with estimates of approximately 100–1,000 CFU-F per milliliter of bone marrow [39], making AT an excellent candidate source for regenerative therapy.

## 4. Characterization of ASCs

Although a minimal set of cell surface markers to be analyzed for MSCs identification has been defined in 2006 [40], the correct immunophenotype characterization of ASCs has been debated for a long time. Due to the inherent SVF heterogeneity, a multiparameter flow cytometric analytic and sorting strategy have been developed. Based on the hematopoietic marker CD45, the endothelial marker CD31, the perivascular marker CD146, and the stem-stromal markers CD34, CD90, CD105, and CD117 (c-kit), four distinct populations have been defined in the SVF fraction in uncultured conditions: putative ASCs (CD31−, CD34+/−, CD45−, CD90+, CD105−, CD117− and CD146−), endothelial-progenitor cells (CD31+, CD34+, CD45−, CD90+, CD105−, CD117+ and CD146+), vascular smooth muscle cells or pericytes (CD31−, CD34+/−, CD45−, CD90+, CD105−, CD117+ and CD146+), and hematopoietic cells (CD45+) [41, 42]. Studies on whole AT have revealed that the stem/progenitor components, organized around small vessels in an annular fashion, are dominated by a prevalent supra-adventitial layer of CD34+ cells displaying MSCs-like multipotentiality [41–43]. These supra-adventitial adipose stromal cells (SA-ASC) surround arterioles and venules, which are colonized on their surfaces by CD146+ perivascular cells or pericytes [42, 44]. A component of proliferative CD34+ and CD31+ endothelial progenitor cells is associated with the luminal layer [45].

Compared to extensively cultivated ASCs, freshly isolated SVF cells and early passage ASCs express higher levels of CD117 (c-kit), human leukocyte antigen-DR, and stem cell-associated markers such as CD34, along with lower levels of stromal cell markers such as CD13, CD29, CD34, CD54, CD73, CD90, CD105, and MHC I [46, 47]. It seems that CD34+ ASCs have a greater proliferative capacity, while CD34− ASCs exert higher plasticity [48, 49].

Recently, the International Federation for Adipose Therapeutics and Science (IFATS) and the International Society for Cellular Therapy (ISCT) have provided initial guidance for the scientific community working with adipose-derived cells defining the minimal criteria for the identification of ASCs [50]. In the SVF, cells are identified by the combination of the following markers: CD45−, CD31−, and CD34+. Added information should be given with the analysis of stromal/stem cell markers: CD13, CD73, CD90, and CD105. In culture, like BM-MSCs, ASCs are positive for CD90, CD73, CD105, and CD44, while negative for CD45 and CD31. Unlike BM-MSCs, ASCs are positive for CD36 and negative for CD106. Finally, to allow the identification of ASCs a multilineage differentiation assay should be performed.

## 5. Osteogenic Potential of ASCs and Their Role in Bone Regeneration

Cell-based approaches for bone formation and regeneration are widely considered the most effective, as they are able to efficiently sustain the physiologic osteogenic process *in vivo*. Indeed, the most promising field for ASCs application is represented by bone reconstruction/regeneration [38, 51]. Bones are dynamic organs, undergoing continuous remodeling to maintain tissue homeostasis, modify shape and morphology, and repair fractures [52]. The therapeutic options clinically available are currently restricted to allografts, microvascular bone, and osteomyocutaneous flaps taken from an autologous donor site, and bone distraction for reconstructive purposes [53–55]. In particular, bone “free flaps” harvested from fibula, scapula, iliac crest, or rib represent the therapeutic gold standard because they contain all the components needed for regeneration, including differentiated bone cells, their cellular precursors, and appropriate growth/differentiation factors. The main disadvantage of this technique relates to the morbidity of the donor site, where a skeletal defect is created. Furthermore, the complexity of autograft procedures raises other technical issues: the maintenance of the arterial and venous flow of the flap in the case of inadequacy of the receiving site (e.g., previous radiation therapy); excessive extension of the bone defect in need of repair; peripheral vasculopathies; and poor general clinical condition [56]. Therefore, scientific research aims to bypass the need for allografts or autologous tissue grafts in repairing large bone defects (either posttraumatic or as a consequence of surgical resection), for which a spontaneous recovery cannot be expected. *In vitro* and *in vivo* models suggest that the use of expanded ASCs improve bone healing through direct differentiation into mature osteoblasts and paracrine effects that facilitate migration and differentiation of resident precursors. The secretome of the SVF [57, 58] and of the ASCs [59, 60] contains different endocrine factors (adipokines) with bone remodeling activity [61–63]. Specifically, the vascular endothelial growth factor (VEGF), present in the secretome of both SVF and ASCs, plays a major role in the repair of fractures or bone defects. The VEGF is able to activate the formation of a new network of blood capillaries, which is required during the physiological process of bone regeneration [64]. In addition, VEGF plays a direct role in the recruitment of hematopoietic stem cells leading to the formation of new bone [65, 66].

The cell osteogenic potential can be assessed *in vitro*, through an induction assay based on a widely standardized protocol, employing a culture medium supplemented with ascorbic acid, dexamethasone, and beta-glycerol phosphate [4]. Thereafter, to verify the acquisition of an osteogenic phenotype, staining protocols are used to detect calcium deposits and matrix mineralization (namely, Von Kossa and alizarin red methods) [67].

## 6. ASCs-Based Gene Therapy Osteoinductive Approaches

In recent years, cell-based osteoinductive gene-delivery techniques have produced the most convincing results both *in vitro* and *in vivo* models. Such methods use cells genetically-engineered to express selected osteogenic factors to be implanted into the anatomical site where bone regeneration is required. To date, recombinant bone morphogenetic proteins (BMPs) have been the most frequently studied and used osteoinducing agents [51, 68–74]. Lately, several new transcription factors involved in the osteogenic process have been reported, including Runx2, vascular endothelial growth factor (VEGF), the LIM mineralization protein (LMP), Sonic Hedgehog (SHH), and Nell-1 [56, 75–81]. In a study performed by Lee and colleagues [75], BMP-2 and RunX2 were coexpressed in ASCs, demonstrating that BMP2/Runx2-ASCs show a significant increase in bone formation compared to ASCs and BMP2-ASCs. Recently, Zhang et al. [77] studied the osteogenic differentiation of ASCs in presence of VEGF, BMP-6, or VEGF plus BMP-6, showing that the combination of VEGF and BMP-6 significantly enhance the expression of osteospecific genes like Dlx5 and osterix and suggesting a cross-talk between VEGF and BMP-6 signaling pathways during the osteogenic differentiation of ASCs. Also, two pro-osteogenic cytokine, Sonic Hedgehog (SHH) and Nell-1, have been studied by James et al. [76], revealing the additive effects of SHH and NELL-1 on the osteogenic differentiation of ASCs.

## 7. Scaffolds for ASCs in Bone Repair

Scaffolds for osteogenesis should mimic bone morphology and structure in order to optimize integration into the surrounding tissue and to provide a suitable microenvironment for MSCs adhesion, proliferation, and differentiation. The micro- and macroarchitecture of the scaffold is known to be highly dependent on the production process [82, 83]. A well-characterized biomaterial is hydroxyapatite (HA), Ca_10_(PO_4_)_6_(OH)_2_, which is currently used in clinical applications in different forms. HA is suitable for substituting or integrating diseased or damaged bone tissues since it resembles the mineralized bone phase and supplies fundamental ions for the newly forming bone during resorption [84, 85]. Also, beta-tricalcium phosphate (*β*-TCP), Ca_3_(PO_4_)_2_, was thought suitable for clinical use as a carrier for MSCs because of its chemical and crystallographic similarities to the inorganic phase of native bone [86, 87]. Biphasic calcium phosphate (BCP) refers to homogenous composites of HA and *β*-TCP [88]. Properties like solubility and resorption capacity of BCP formulations vary widely among different ratios of HA and *β*-TCP. Unfortunately, calcium phosphate ceramics tend to have poor mechanical properties, predisposing them to brittleness and fractures [89, 90]. In the last years, several *in vitro* and *in vivo* studies highlight the osteoinductive role of biomimetic scaffold on ASCs [91, 92]. In particular, a study performed by Marino and collaborators [92] revealed that *β*-TCP matrix alone is sufficient to trigger the differentiation of ASCs toward an osteoblastic phenotype, regardless of whether the cells are grown in a proliferative or a differentiative medium. Also, Liao et al. [91] compared the osteogenic potential of porcine ASCs (P-ASCs) among three scaffold (polycaprolactone, PCL; polycaprolactone and *β*-tricalcium phosphate, PCL-TCP; collagen I coated-PCL-TCP, PCL-TCP-COL), in order to find an optimal scaffold for bone tissue engineering. The *in vitro* study demonstrated that pASCs display the best osteogenic differentiation rate on PCL-TCP-COL group scaffolds, as demonstrated by the highest ALP activity, osteocalcin expression and mineralization [91]. Also, the experiment in nude mice showed better woven bone and vascular tissue formation in the PCL-TCP-COL group than in the PCL group. In addition, the osteogenic ability of pASCs was found to be enhanced by coating COL onto the PCL-TCP scaffolds, both *in vitro* and *in vivo*. Moreover, Arrigoni et al. [93] compared the neoformed bone tissues achieved by treating critical tibial defects with either hydroxyapatite alone (HA, group I) or hydroxyapatite–autologous ASC constructs (ASCs-HA, group II), investigating their histomorphometric, immunohistochemical, and biomechanical properties. The study displayed that tibial defects treated with rabbit ASCs-HA showed an improved healing process when compared to naked scaffold-treated ones [93].

Calcium-, magnesium-, and silicon-containing ceramics, such as akermanite (Ca_2_MgSi_2_O_7_), show better mechanical properties and degradation rates than other bioceramics and are reported to enhance osteogenic commitment of MSCs [86, 87, 94–96]. As shown by Liu and colleagues, human ASCs attachment and proliferation were similar on akermanite and *β*-TCP *in vitro*, and osteogenic ASCs differentiation was enhanced on the akermanite over the *β*-TCP after 10 days of culture [86]. Recently, Zanetti and collegaues observed that ASCs cultured for 21 days in osteogenic medium prior to be seeded onto akermanite-based scaffolds produce greater calcium deposition and osteocalcin expression, compared to cells seeded on *β*-TCP and PCL [94].

Taken together, these data highlight the advantage of using ASCs in combination with biomimetic scaffold providing a most effective strategy for treating bone defects. 

## 8. Dynamic Culture Systems for Cell-Scaffold Constructs

Tissue formation in three-dimensional scaffolds is significantly affected by nutrient transport, physical stress, cell density, and gas exchange [97, 98]. For the best possible tissue regeneration, postimplantation cell viability and homogenous cell distribution throughout the scaffold are crucial [99]. Dynamic systems like perfusion bioreactors facilitate optimal seeding under controlled conditions [99]. The term “bioreactor” refers to a wide variety of culture systems that provide a mechanism to maintain cell-scaffold constructs in a biocompatible environment during application of defined chemical and physical stimuli. Perfusion bioreactors are culture systems in which nutrient medium is repeatedly forced or “perfused” through cell-scaffold constructs. Therefore, these are referred to as “dynamic” culture systems in order to distinguish them from “static” cultures in which there is no fluid motion (i.e., standard culture flask or plate). Such culturing systems are aimed at allowing tridimensional cell adhesion on the scaffold and inducing specific cell behavior under controlled and repeatable conditions. This situation mimics a complex natural environment, as the cell-scaffold compound is exposed to common mechanical stimuli, deriving from the shear forces from nutrient medium motion and enables generating constructs with increased functionality and engraftment capacity [99, 100].

So far, few studies have described the possibility to establish a 3D culture model for bone cells using mineralized porous scaffolds as templates, which relies on the use of a perfusion-based bioreactor device, highlighting the synergism between a bioactive scaffold and the effect of perfusion on cells and indicating the differentiation into an osteogenic phenotype [100, 101]. In particular, in the study by Fröhlich and collaborators [102] ASCs were seeded on decellularized native bone scaffolds, providing the necessary structural and mechanical environment for osteogenic differentiation, and cultured in a perfusion bioreactor. After 5 weeks of culture, the addition of osteogenic supplements (dexamethasone, sodium-beta-glycerophosphate, and ascorbic acid-2-phosphate) to culture medium significantly increased the construct cellularity and the amounts of bone matrix components (collagen, bone sialoprotein, and bone osteopontin), indicating that medium perfusion markedly improved the distribution of cells and bone matrix in engineered constructs [102]. Also, in the study performed by Declercq and colleagues [103], After 6 weeks of dynamic culture, scaffolds were highly colonized and the osteogenic gene expression was higher compared to static cultures. Recently, Silva and colleagues [104] demonstrated that ASCs differentiate towards the osteogenic phenotype when cultured in a bioactive glass scaffold, with the osteogenic Leibovitz L-15 medium and a perfusion bioreactor, as indicated both the significant increase in cell proliferation and viability, the increased ALP activity, and the expression of osteospecific protein (i.e., osteocalcin and osteopontin) 2-to-3 weeks after culture. Furthermore, a coculture model of human osteoblast and endothelial lineage cells has been established by seeding and culturing cells freshly isolated from the SVF of AT within porous 3D ceramic scaffolds [105]. This system was reported to generate 3D constructs that, upon implantation into nude mice, were able to generate bone tissue and fully functional blood vessels [105, 106]. Also, a study performed by Güven and colleagues [107] remarks the efficiency of SVF cells to generate 3D-osteogenic constructs, compared to ASCs, supporting the concept that vascular progenitors derived from human SVF cells accelerate the engraftment of critically sized osteogenic constructs, ultimately improving the efficiency and uniformity of bone tissue formation.

## 9. Preclinical Evaluation of ASC Osteoregenerative Potential

A huge amount of data in the literature demonstrates the efficacy of ASC-based approaches for inducing bone regeneration/healing *in vivo*. Critical size-calvarial defects are widely employed to study bone healing in animal models, mostly rodents, allowing an easy quantification of the amount of newly formed bone within a bidimensional defect [74, 108–127]. An initial proof of principle of the *in vivo* osteogenic potential of experimental constructs may be achieved using local intramuscular injection to induce ectopic bone formation [75, 121, 128–133]. Also, segmental defects in long bones of large animals are widely used as clinically relevant models, as resembling the fracture healing process [93, 112, 134–147]. 

A number of published report indicates that the combination of recombinant human BMP2 (rhBMP2) with ASC may increase the osteogenic potential *in vivo* (see Table 1), although recent evidences are retracting this consolidated dogma, suggesting that combining rhBMP2 with ASCs, should not be considered the best viable strategy for inducing bone healing. 

Overall, the number of published data obtained from animal models employed to study the bone healing properties of ASCs is constantly growing. Although a comprehensive and systematic categorization of all publications on this topic may be quite impossible, Table 1 attempts to summarize the study design of relevant preclinical studies. It is noteworthy that successful results, in terms of bone healing, have been achieved in different animal models, using either undifferentiated ASC (i.e., in the absence of any prior *ex vivo* osteogenic induction) [93, 111, 114–116, 125, 128, 131, 136, 138, 140, 145–147] or uncultured SVF [112, 123] paving the way to an easier translation of preclinical evidence to the clinical setting.

## 10. Clinical Use of ASCs for Bone Regeneration/Reconstruction

When attempting to translate preclinical evidence to the clinical field, the manipulation of human tissues, for the production of clinical-grade human SVF cells and ASCs to be employed as therapeutic devices, must be carried out according to the current good manufacturing practices (GMP). The national regulatory agencies (i.e., the Food and Drug Administration in USA and the European Medicines Agency in EU) provide the official rules and guidelines that guarantee safe and controlled procedures [148]. In particular, the SVF should be classified as a minimally manipulated tissue, whose isolation does not require seeding and culturing. Conversely, all procedures involving culture-expanded MSC configure advanced cell therapies and must comply with institutional GMP rules for cell manipulation, which must be carried out into a cell factory of a certified facility. 

In recent years, ASCs attracted the overwhelming interest of clinicians and industry, being multipotent stem cells endowed with trophic and immune-modulatory properties, residing into a widely available and relatively accessible adult tissue. This has been generating a confusing scenario that often risks to configure clinical misconduct, when putative innovative cell therapies are provided within uncontrolled trials to incorrectly informed patients, in a wide range of clinical applications. On this regard, a useful lesson for “naive” clinicians may be provided by the controversial debate, recently brought by Italian media, around the “Stamina Foundation,” which promoted the use of bone marrow-derived MSCs as a “compassionate, as yet-unapproved” treatment of neurodegenerative diseases (including spinal muscular atrophy) in terminally ill children [149]. After all, the proposed protocol for MSC processing, the so called “Vannoni's method,” was carried out in inappropriate facilities (according to the Italian Medicines Agency, AIFA) and was based on flawed and plagiarized data [150]. 

In bone reconstructive surgery, autologous or allogeneic bone graft still represents the gold standard treatment although hampered by local morbidity and largely relying on donor availability, expecially in the case of large segments to be harvested. Therefore, the need for alternative procedure has rapidly lead to experimental procedures based on ASCs. Despite the increasing amount of scientific data on ASCs and an extremely wide number of preclinical studies confirming their bone regenerative potential *in vivo*, only few controlled clinical trials, aimed at assessing the efficacy and safety of ASCs in patients with bone-related disorders, have been concluded and published (for review see [148] and [151]) and few others are being currently carried out (http://www.clinicaltrials.gov/). In particular, successful results have been obtained in distinct trials using autologous ASC for craniofacial bone reconstruction [149, 152–154].

Lendeckel and colleagues employed ASC for the reconstruction of a large pediatric posttraumatic calvarial defect, which is always challenging and troublesome. In this case, an interdisciplinary surgical equipe implanted a resorbable macroporous sheet as a scaffold for ASC and milled autograft cancellous bone. The complex procedures yielded a stable osteointegrated graft that showed marked ossification at the 3 months followup [153]. Also Thesleff and collaborators used ASCs for calvarial reconstruction, testing alternative biomaterials (*β*-TCP and resorbable mesh bilaminate scaffold), and obtaining successful results in adult patients [152]. Mesimäki and colleagues used autologous ASCs seeded on a beta-tricalcium phosphate (*β*-TCP) scaffold doped with recombinant human BMP2 to treat a large maxillary defect resulting from a benign tumor resection in an adult patient. They achieved satisfactory outcomes, obtaining new, mature, vital, and vascularized bone eight months after surgery, with good osteointegration and stability [154]. More recently, Sandor and colleagues published the successful reconstruction of large anterior mandibular bone defects using ASC seeded on a *β*-TCP premolded scaffold based on patient's computed tomography data [149].

The partial drawbacks of experimental ASC-based bone reconstructive procedures are represented by the need to expand cells *ex vivo* for two-to-three weeks to achieve the appropriate cellular yield prior to the implantation, which implies multiple surgical interventions. Moreover, extended *in vitro* ASC expansion may be associated to genomic instability leading to either structural or numeric chromosomal aberrations [155], though it is still unclear whether this may represent a real risk for the recipient patient. Recent research efforts have been spent to develop *ad hoc* devices for the rapid one-step isolation of the SVF from liposuctioned adipose tissue to be grafted without prior *ex vivo* culture amplification manipulation [156]. Further development of such devices may allow overcoming and implementing fat harvesting for ASC isolation aimed at reconstructive surgery.

## 11. Conclusions

Around 3000 publication surveyed in the scientific databases point towards the definition of ASCs as the most effective and safe cell type for regenerative medicine approaches. Bone regeneration is currently the most promising field for clinical translation of experimental ASCs protocols. Nonetheless, the rapidly growing development of research in the field of biocompatible scaffolds is widening the field of ASCs applications in multidisciplinary scenarios, allowing cells to grow, differentiate, and be exposed to cytokines and growth factors.

## Figures and Tables

**Table 1:** Preclinical studies on ASC osteoregenerative potential.

Experimental model	Species	Scaffold/administration	Additional *ex vivo*/*in vivo* treatment	Graft type	Reference
Calvarial defect	Rat	PLGA	Alendronate	Xenogeneic	[74]
	Rabbit	HA-PLGA, collagen sponge	BV-BMP2/TGF *β*3	Allogeneic	[120]
	Mouse	PLGA	Dura mater	Xenogeneic	[117]
	Rat	*β*-TCP	Lenti-miR-31	Allogeneic	[110]
	Mouse	Custom scaffold	NOGGIN shRNA-Knockout	Xenogeneic	[119]
	Dog	HA-PLGA	None	Xenogeneic	[122]
	Mouse	Systemic injection	None	Allo/xenogeneic	[115]
	Mouse	Local injection	None	Xenogeneic	[116]
	Rat	DBM, PLA	None	Xenogeneic	[123]
	Rat	MAP-coated PCL/PLGA	None	Xenogeneic	[111]
	Rat	HA-*β*-TCP	None	Xenogeneic	[126]
	Rat	PLGA	None/osteogenic medium	Xenogeneic	[125]
	Dog	Coral	Osteogenic induction	Autologous	[109]
	Dog	Coral	Osteogenic induction	Allogeneic	[121]
	Pig	Collagen sponge	Osteogenic induction	Autologous	[127]
	Rat	DBX	Osteogenic induction	Allogeneic	[112]
	Rat	PCL-PLGA-*β*-TCP	Osteogenic induction + HUVEC	Xenogeneic	[113]
	Mouse	pDA-PLGA	rhBMP-2	Xenogeneic	[114]
	Rabbit	Collagen sponge	rhBMP-2	Allogeneic	[124]
	Mouse	HA-PLGA	Sonic hedgehog signaling Induction	Xenogeneic	[118]
	Rat	Local injection	VEGFa	Xenogeneic	[108]

Ectopic bone formation	Mouse	PLGA	BMP2/RUNX2 bicistronic vector	Xenogeneic	[75]
	Mouse	PRP + alginate microsphere	None	Allogeneic	[131]
	Mouse	*β*-TCP	None	Xenogeneic	[121]
	Rat	HA	None	Xenogeneic	[128]
	Rat	Matrigel	Osteogenic induction	Xenogeneic	[133]
	Rat	DBM	Osteogenic induction	Xenogeneic	[132]
	Mouse	Carbon nanotubes	rhBMP2	Xenogeneic	[130]
	Rat	PLDA	rhBMP2	Xenogeneic	[129]

Segmental defect	Rabbit	Local injection	Bovine BMP	Allogeneic	[135]
	Rat	Fibrin matrix	rhBMP2	Allogeneic	[139]
	Rat	*β*-TCP	Lenti-BMP2/7	Allogeneic	[134]
	Rabbit	PLA/PCL + vascularized periosteum	Ad-Cbfa1	Allogeneic	[140]
	Rabbits	HA-PLA-COL	Ad-hBMP2	Allogeneic	[137]

Segmental defect	Mouse	Systemic injection	None	Allogeneic	[140]
	Rat	Collagen gel	None	Xenogeneic	[145]
	Rabbit	PLGA	None/osteogenic medium	Xenogeneic	[112]
	Dog	*β*-TCP	None	Allogeneic	[138]
	Rabbit	HA	None	Autologous	[93]
	Rabbit	Ceramics, biphasic materials	None	Allogeneic	[136]

Vertebral defect/fusion	Mouse	Local injection	rhBMP6 nucleofection	Xenogeneic	[143]
	Rat	Lyophilized human cancellous bone	Gal-KO + osteogenic induction	Xenogeneic	[142]
	Rat	Fibrin gel	rhBMP6 nucleofection	Xenogeneic	[144]

Mandible defect	Pig	Local-systemic injection	None	Allogeneic	[147]
	Rat	HA/COL	None	Xenogeneic	[146]

HA: hydroxyapatite; PLGA: poly(lactic-co-glycolic acid); PLA/PCL: polylactic acid/polycaprolacton; Ad-Cbfa1: adenoviral expression vector carrying the Cbfa1 gene; DBM: demineralized bone matrix; *β*-TCP: beta-tricalcium phosphate; Lenti-miR-31: lentivirus expression vector carrying the microRNA-31; p-DA: polydopamine; PRP: platelet-rich plasma; Lenti-BMP2/7: lentivirus expression vector carrying either the BMP2 or the BMP7 gene, MAP: mussel adhesive proteins, NOGGIN shRNA: short hairpin ribonucleic acid to knockdown NOGGIN gene, COL: collagen; BV-BMP2/TGF *β*3: baculovirus expression vector carrying either the BMP2 or the TGF *β*3 gene, MAP: mussel adhesive proteins; Gal-KP: galactosyl-knock-out; a-CaP: amorphous calcium phosphate; *these studies were based on uncultured SVF instead of culture-amplified ASCs.

## References

[1] Friedenstein AJ (1961). Osteogenetic activity of transplanted transitional epithelium. *Acta anatomica*.

[2] Saulnier N, Puglisi MA, Lattanzi W (2011). Gene profiling of bone marrow- and adipose tissue-derived stromal cells: a key role of Kruppel-like factor 4 in cell fate regulation. *Cytotherapy*.

[3] Liu AR, Liu L, Chen S (2013). Activation of canonical wnt pathway promotes differentiation of mouse bone marrow-derived MSCs into type II alveolar epithelial cells, confers resistance to oxidative stress, and promotes their migration to injured lung tissue in vitro. *Journal of Cellular Physiology*.

[4] Saulnier N, Lattanzi W, Puglisi MA (2009). Mesenchymal stromal cells multipotency and plasticity: induction toward the hepatic lineage. *European Review for Medical and Pharmacological Sciences*.

[5] Saulnier N, Piscaglia AC, Puglisi MA (2010). Molecular mechanisms underlying human adipose tissue-derived stromal cells differentiation into a hepatocyte-like phenotype. *Digestive and Liver Disease*.

[6] Puglisi MA, Saulnier N, Piscaglia AC, Tondi P, Agnes S, Gasbarrini A (2011). Adipose tissue-derived mesenchymal stem cells and hepatic differentiation: old concepts and future perspectives. *European Review for Medical and Pharmacological Sciences*.

[7] Puglisi MA, Tesori V, Lattanzi W (2011). Therapeutic implications of mesenchymal stem cells in liver injury. *Journal of Biomedicine and Biotechnology*.

[8] Yabana T, Arimura Y, Tanaka H (2009). Enhancing epithelial engraftment of rat mesenchymal stem cells restores epithelial barrier integrity. *Journal of Pathology*.

[9] Valcz G, Krenács T, Sipos F (2011). The role of the bone marrow derived mesenchymal stem cells in colonic epithelial regeneration. *Pathology and Oncology Research*.

[10] Bussolati B, Tetta C, Camussi G (2008). Contribution of stem cells to kidney repair. *American Journal of Nephrology*.

[11] Neirinckx V, Marquet A, Coste C, Rogister B, Wislet-Gendebien S (2013). Adult bone marrow neural crest stem cells and mesenchymal stem cells are not able to replace lost neurons in acute MPTP-lesioned mice. *PLoS ONE*.

[12] Hu F, Wang X, Liang G (2013). Effects of epidermal growth factor and basic fibroblast growth factor on the proliferation and osteogenic and neural differentiation of adipose-derived stem cells. *Cellular Reprogramming*.

[13] Bai WF, Xu WC, Feng Y (2013). Fifty-Hertz electromagnetic fields facilitate the induction of rat bone mesenchymal stromal cells to differentiate into functional neurons. *Cytotherapy*.

[14] Wislet-Gendebien S, Wautier F, Leprince P, Rogister B (2005). Astrocytic and neuronal fate of mesenchymal stem cells expressing nestin. *Brain Research Bulletin*.

[15] Cai S, Shea GKH, Tsui AYP, Chan YS, Shum DKY (2011). Derivation of clinically applicable schwann cells from bone marrow stromal cells for neural repair and regeneration. *CNS and Neurological Disorders*.

[16] Pan Y, Cai S (2012). Current state of the development of mesenchymal stem cells into clinically applicable Schwann cell transplants. *Molecular and Cellular Biology*.

[17] Lattanzi W, Geloso MC, Saulnier N (2011). Neurotrophic features of human adipose tissue-derived stromal cells: in vitro and in vivo studies. *Journal of Biomedicine and Biotechnology*.

[18] Dupin E, Sommer L (2012). Neural crest progenitors and stem cells: from early development to adulthood. *Developmental Biology*.

[19] Moroni L, Fornasari PM (2013). Human mesenchymal stem cells: a bank perspective on the isolation, characterization and potential of alternative sources for the regeneration of musculoskeletal tissues. *Journal of Cellular Physiology*.

[20] de Coppi P, Bartsch G, Siddiqui MM (2007). Isolation of amniotic stem cell lines with potential for therapy. *Nature Biotechnology*.

[21] Barba M, Pirozzi F, Saulnier N (2012). Lim mineralization protein 3 induces the osteogenic differentiation of human amniotic fluid stromal cells through Kruppel-like factor-4 downregulation and further bone-specific gene expression. *Journal of Biomedicine and Biotechnology*.

[22] Chao YH, Wu HP, Chan CK, Tsai C, Peng CT, Wu KH (2012). Umbilical cord-derived mesenchymal stem cells for hematopoietic stem cell transplantation. *Journal of Biomedicine and Biotechnology*.

[23] Longo UG, Loppini M, Berton A, La VL, Khan WS, Denaro V (2012). Stem cells from umbilical cord and placenta for musculoskeletal tissue engineering. *Current Stem Cell Research & Therapy*.

[24] Yang S, Huang S, Feng C, Fu X (2012). Umbilical cord-derived mesenchymal stem cells: strategies, challenges, and potential for cutaneous regeneration. *Frontiers of Medicine*.

[25] Peroni D, Scambi I, Pasini A (2008). Stem molecular signature of adipose-derived stromal cells. *Experimental Cell Research*.

[26] Yoshimura K, Suga H, Eto H (2009). Adipose-derived stem/progenitor cells: roles in adipose tissue remodeling and potential use for soft tissue augmentation. *Regenerative Medicine*.

[27] Weisberg SP, McCann D, Desai M, Rosenbaum M, Leibel RL, Ferrante AW (2003). Obesity is associated with macrophage accumulation in adipose tissue. *Journal of Clinical Investigation*.

[28] Xu H, Barnes GT, Yang Q (2003). Chronic inflammation in fat plays a crucial role in the development of obesity-related insulin resistance. *Journal of Clinical Investigation*.

[29] Prunet-Marcassus B, Cousin B, Caton D, André M, Pénicaud L, Casteilla L (2006). From heterogeneity to plasticity in adipose tissues: site-specific differences. *Experimental Cell Research*.

[30] Guercio A, Di BS, Casella S, Di MP, Russo C, Piccione G (2013). Canine mesenchymal stem cells (MSCs): characterization in relation to donor age and adipose tissue-harvesting site. *Cell Biology International*.

[31] Requicha JF, Viegas CA, Albuquerque CM, Azevedo JM, Reis RL, Gomes ME (2012). Effect of anatomical origin and cell passage number on the stemness and osteogenic differentiation potential of canine adipose-derived stem cells. *Stem Cell Reviews and Reports*.

[32] von Eyben FE, Kroustrup JP, Larsen JF, Celis J (2004). Comparison of gene expression in intra-abdominal and subcutaneous fat: a study of men with morbid obesity and nonobese men using microarray and proteomics. *Annals of the New York Academy of Sciences*.

[33] Grove KL, Fried SK, Greenberg AS, Xiao XQ, Clegg DJ (2010). A microarray analysis of sexual dimorphism of adipose tissues in high-fat-diet-induced obese mice. *International Journal of Obesity*.

[34] Linder K, Arner P, Flores-Morales A, Tollet-Egnell P, Norstedt G (2004). Differentially expressed genes in visceral or subcutaneous adipose tissue of obese men and women. *Journal of Lipid Research*.

[35] Seale P, Bjork B, Yang W (2008). PRDM16 controls a brown fat/skeletal muscle switch. *Nature*.

[36] Mizuno H, Tobita M, Uysal AC (2012). Concise review: adipose-derived stem cells as a novel tool for future regenerative medicine. *Stem Cells*.

[37] Sterodimas A, de Faria J, Nicaretta B, Pitanguy I (2010). Tissue engineering with adipose-derived stem cells (ADSCs): current and future applications. *Journal of Plastic, Reconstructive and Aesthetic Surgery*.

[38] Lindroos B, Suuronen R, Miettinen S (2011). The potential of adipose stem cells in regenerative medicine. *Stem Cell Reviews and Reports*.

[39] Strem BM, Hicok KC, Zhu M (2005). Multipotential differentiation of adipose tissue-derived stem cells. *Keio Journal of Medicine*.

[40] Dominici M, Le Blanc K, Mueller I (2006). Minimal criteria for defining multipotent mesenchymal stromal cells. The International Society for Cellular Therapy position statement. *Cytotherapy*.

[41] Lin K, Matsubara Y, Masuda Y (2008). Characterization of adipose tissue-derived cells isolated with the Celution system. *Cytotherapy*.

[42] Zimmerlin L, Donnenberg VS, Pfeifer ME (2010). Stromal vascular progenitors in adult human adipose tissue. *Cytometry Part A*.

[43] Corselli M, Chen CW, Sun B, Yap S, Rubin JP, Peault B (2012). The tunica adventitia of human arteries and veins as a source of mesenchymal stem cells. *Stem Cells and Development*.

[44] Zimmerlin L, Donnenberg VS, Donnenberg AD (2012). Pericytes: a universal adult tissue stem cell?. *Cytometry Part A*.

[45] Zimmerlin L, Donnenberg VS, Rubin JP, Donnenberg AD (2013). Mesenchymal markers on human adipose stem/progenitor cells. *Cytometry A*.

[46] Gronthos S, Franklin DM, Leddy HA, Robey PG, Storms RW, Gimble JM (2001). Surface protein characterization of human adipose tissue-derived stromal cells. *Journal of Cellular Physiology*.

[47] Mitchell JB, McIntosh K, Zvonic S (2006). Immunophenotype of human adipose-derived cells: temporal changes in stromal-associated and stem cell-associated markers. *Stem Cells*.

[48] Bailey AM, Kapur S, Katz AJ (2010). Characterization of adipose-derived stem cells: an update. *Current Stem Cell Research and Therapy*.

[49] Suga H, Matsumoto D, Eto H (2009). Functional implications of CD34 expression in human adipose-derived stem/progenitor cells. *Stem Cells and Development*.

[50] Bourin P, Bunnell BA, Casteilla L (2013). Stromal cells from the adipose tissue-derived stromal vascular fraction and culture expanded adipose tissue-derived stromal/stem cells: a joint statement of the International Federation for Adipose Therapeutics and Science (IFATS) and the International Society for Cellular Therapy (ISCT). *Cytotherapy*.

[51] Lattanzi W, Pola E, Pecorini G, Logroscino CA, Robbins PD (2005). Gene therapy for in vivo bone formation: recent advances. *European Review for Medical and Pharmacological Sciences*.

[52] Lattanzi W, Bernardini C, Lin Y (2011). Genes and molecular pathways of the osteogenic process. *Osteogenesis*.

[53] Albertson KS, Medoff RJ, Mitsunaga MM (1991). The use of periosteally vascularized autografts to augment the fixation of large segmental allografts. *Clinical Orthopaedics and Related Research*.

[54] Enneking WF, Mindell ER (1991). Observations on massive retrieved human allografts. *Journal of Bone and Joint Surgery A*.

[55] Borden M, Attawia M, Khan Y, El-Amin SF, Laurencin CT (2004). Tissue-engineered bone formation in vivo using a novel sintered polymeric microsphere matrix. *Journal of Bone and Joint Surgery B*.

[56] Parrilla C, Lattanzi W, Fetoni AR, Bussu F, Pola E, Paludetti G (2010). Ex vivo gene therapy using autologous dermal fibroblasts expressing hLMP3 for rat mandibular bone regeneration. *Head and Neck*.

[57] He J, Duan H, Xiong Y (2013). Participation of CD34-enriched mouse adipose cells in hair morphogenesis. *Molecular Medicine Reports*.

[58] de Francesco F, Tirino V, Desiderio V (2009). Human CD34+/CD90+ ASCs are capable of growing as sphere clusters, producing high levels of VEGF and forming capillaries. *PLoS ONE*.

[59] Kapur SK, Katz AJ (2013). Review of the adipose derived stem cell secretome. *Biochimie*.

[60] Kilroy GE, Foster SJ, Wu X (2007). Cytokine profile of human adipose-derived stem cells: expression of angiogenic, hematopoietic, and pro-inflammatory factors. *Journal of Cellular Physiology*.

[61] Ducy P (2011). The role of osteocalcin in the endocrine cross-talk between bone remodelling and energy metabolism. *Diabetologia*.

[62] Gómez-Ambrosi J, Rodríguez A, Catalán V, Frühbeck G (2008). The bone-adipose axis in obesity and weight loss. *Obesity Surgery*.

[63] Reid IR (2008). Relationships between fat and bone. *Osteoporosis International*.

[64] Colnot C (2005). Cellular and molecular interactions regulating skeletogenesis. *Journal of Cellular Biochemistry*.

[65] Ferrara N (2004). Vascular endothelial growth factor: basic science and clinical progress. *Endocrine Reviews*.

[66] Peng H, Usas A, Olshanski A (2005). VEGF improves, whereas sFlt1 inhibits, BMP2-induced bone formation and bone healing through modulation of angiogenesis. *Journal of Bone and Mineral Research*.

[67] Lattanzi W, Barba M, Novegno F (2013). Lim mineralization protein is involved in the premature calvarial ossification in sporadic craniosynostoses. *Bone*.

[68] Cook SD, Wolfe MW, Salkeld SL, Rueger DC (1995). Effect of recombinant human osteogenic protein-1 on healing of segmental defects in non-human primates. *Journal of Bone and Joint Surgery A*.

[69] Einhorn TA (2003). Clinical applications of recombinant human BMPs: early experience and future development. *Journal of Bone and Joint Surgery A*.

[70] Groeneveld EHJ, Burger EH (2000). Bone morphogenetic proteins in human bone regeneration. *European Journal of Endocrinology*.

[71] Cowan CM, Aalami OO, Shi YY (2005). Bone morphogenetic protein 2 and retinoic acid accelerate in vivo bone formation, osteoclast recruitment, and bone turnover. *Tissue Engineering*.

[72] Dragoo JL, Choi JY, Lieberman JR (2003). Bone induction by BMP-2 transduced stem cells derived from human fat. *Journal of Orthopaedic Research*.

[73] Miyazaki M, Zuk PA, Zou J (2008). Comparison of human mesenchymal stem cells derived from adipose tissue and bone marrow for ex vivo gene therapy in rat spinal fusion model. *Spine*.

[74] Wang CZ, Chen SM, Chen CH (2010). The effect of the local delivery of alendronate on human adipose-derived stem cell-based bone regeneration. *Biomaterials*.

[75] Lee SJ, Kang SW, Do HJ (2010). Enhancement of bone regeneration by gene delivery of BMP2/Runx2 bicistronic vector into adipose-derived stromal cells. *Biomaterials*.

[76] James AW, Pang S, Askarinam A (2012). Additive effects of sonic hedgehog and Nell-1 signaling in osteogenic versus adipogenic differentiation of human adipose-derived stromal cells. *Stem Cells and Development*.

[77] Zhang Y, Madhu V, Dighe AS, Irvine JN, Cui Q (2012). Osteogenic response of human adipose-derived stem cells to BMP-6, VEGF, and combined VEGF plus BMP-6 in vitro. *Growth Factors*.

[78] Boden SD, Titus L, Hair G (1998). 1998 Volvo award winner in basic sciences studies: lumbar spine fusion by local gene therapy with a cDNA encoding a novel osteoinductive protein (LMP-1). *Spine*.

[79] Pola E, Gao W, Zhou Y (2004). Efficient bone formation by gene transfer of human LIM mineralization protein-3. *Gene Therapy*.

[80] Bernardini C, Saulnier N, Parrilla C (2010). Early transcriptional events during osteogenic differentiation of human bone marrow stromal cells induced by Lim mineralization protein 3. *Gene Expression*.

[81] Lattanzi W, Parrilla C, Fetoni A (2008). Ex vivo-transduced autologous skin fibroblasts expressing human Lim mineralization protein-3 efficiently form new bone in animal models. *Gene Therapy*.

[82] Martinetti R, Dolcini L, Belpassi A (2004). Inspired porosity for cells and tissues. *Key Engineering Materials*.

[83] Tampieri A, Celotti G, Landi E (2005). From biomimetic apatites to biologically inspired composites. *Analytical and Bioanalytical Chemistry*.

[84] Dorozhkin SV, Epple M (2002). Biological and medical significance of calcium phosphates. *Angewandte Chemie*.

[85] Schmitz JP, Hollinger JO, Milam SB (1999). Reconstruction of bone using calcium phosphate bone cements: a critical review. *Journal of Oral and Maxillofacial Surgery*.

[86] Liu Q, Cen L, Yin S (2008). A comparative study of proliferation and osteogenic differentiation of adipose-derived stem cells on akermanite and *β*-TCP ceramics. *Biomaterials*.

[87] Hing KA, Wilson LF, Buckland T (2007). Comparative performance of three ceramic bone graft substitutes. *Spine Journal*.

[88] Kruyt MC, Wilson CE, de Bruijn JD (2006). The effect of cell-based bone tissue engineering in a goat transverse process model. *Biomaterials*.

[89] Petite H, Viateau V, Bensaïd W (2000). Tissue-engineered bone regeneration. *Nature Biotechnology*.

[90] Lopez MJ, Daigle PR (2013). Adult multipotent stromal cell technology for bone regeneration: a review. *Veterinary Surgery*.

[91] Liao HT, Lee MY, Tsai WW, Wang HC, Lu WC (2013). Osteogenesis of adipose-derived stem cells on polycaprolactone-beta-tricalcium phosphate scaffold fabricated via selective laser sintering and surface coating with collagen type I. *Journal of Tissue Engineering and Regenerative Medicine*.

[92] Marino G, Rosso F, Cafiero G (2010). *β*-Tricalcium phosphate 3D scaffold promote alone osteogenic differentiation of human adipose stem cells: in vitro study. *Journal of Materials Science: Materials in Medicine*.

[93] Arrigoni E, de GL, Di GA (2013). Adipose-derived stem cells and rabbit bone regeneration: histomorphometric, immunohistochemical and mechanical characterization. *Journal of Orthopaedic Science*.

[94] Zanetti A, McCandless G, Chan J, Gimble J, Hayes D (2012). Characterization of novel akermanite:poly-epsilon-caprolactone scaffolds for human adipose-derived stem cells bone tissue engineering. *Journal of Tissue Engineering and Regenerative Medicine*.

[95] Gu H, Guo F, Zhou X (2011). The stimulation of osteogenic differentiation of human adipose-derived stem cells by ionic products from akermanite dissolution via activation of the ERK pathway. *Biomaterials*.

[96] Zanetti AS, McCandless GT, Chan JY, Gimble JM, Hayes DJ (2013). In vitro human adipose-derived stromal/stem cells osteogenesis in akermanite: poly-*ε*-caprolactone scaffolds. *Journal of Biomaterials Applications*.

[97] Peng CA, Palsson BO (1996). Determination of specific oxygen uptake rates in human hematopoietic cultures and implications for bioreactor design. *Annals of Biomedical Engineering*.

[98] Cancedda R, Giannoni P, Mastrogiacomo M (2007). A tissue engineering approach to bone repair in large animal models and in clinical practice. *Biomaterials*.

[99] Janssen FW, Hofland I, van Oorschot A, Oostra J, Peters H, van Blitterswijk CA (2006). Online measurement of oxygen consumption by goat bone marrow stromal cells in a combined cell-seeding and proliferation perfusion bioreactor. *Journal of Biomedical Materials Research A*.

[100] Wang Y, Uemura T, Dong J, Kojima H, Tanaka J, Tateishi T (2003). Application of perfusion culture system improves in vitro and in vivo osteogenesis of bone marrow-derived osteoblastic cells in porous ceramic materials. *Tissue Engineering*.

[101] Wang C, Cen L, Yin S (2010). A small diameter elastic blood vessel wall prepared under pulsatile conditions from polyglycolic acid mesh and smooth muscle cells differentiated from adipose-derived stem cells. *Biomaterials*.

[102] Fröhlich M, Grayson WL, Marolt D, Gimble JM, Kregar-Velikonja N, Vunjak-Novakovic G (2010). Bone grafts engineered from human adipose-derived stem cells in perfusion bioreactor culture. *Tissue Engineering A*.

[103] Declercq HA, De CT, Krysko O, Bachert C, Cornelissen MJ (2013). Bone grafts engineered from human adipose-derived stem cells in dynamic 3D-environments. *Biomaterials*.

[104] Silva AR, Paula AC, Martins TM, Goes AM, Pereria MM (2013). Synergistic effect between bioactive glass foam and a perfusion bioreactor on osteogenic differentiation of human adipose stem cells. *Journal of Biomedical Materials Research Part A*.

[105] Scherberich A, Galli R, Jaquiery C, Farhadi J, Martin I (2007). Three-dimensional perfusion culture of human adipose tissue-derived endothelial and osteoblastic progenitors generates osteogenic constructs with intrinsic vascularization capacity. *Stem Cells*.

[106] Papadimitropoulos A, Scherberich A, Güven S (2011). A 3D in vitro bone organ model using human progenitor cells. *European Cells & Materials*.

[107] Güven S, Mehrkens A, Saxer F (2011). Engineering of large osteogenic grafts with rapid engraftment capacity using mesenchymal and endothelial progenitors from human adipose tissue. *Biomaterials*.

[108] Behr B, Tang C, Germann G, Longaker MT, Quarto N (2011). Locally applied vascular endothelial growth factor A increases the osteogenic healing capacity of human adipose-derived stem cells by promoting osteogenic and endothelial differentiation. *Stem Cells*.

[109] Cui L, Liu B, Liu G (2007). Repair of cranial bone defects with adipose derived stem cells and coral scaffold in a canine model. *Biomaterials*.

[110] Deng Y, Zhou H, Zou D (2013). The role of miR-31-modified adipose tissue-derived stem cells in repairing rat critical-sized calvarial defects. *Biomaterials*.

[111] Hong JM, Kim BJ, Shim JH (2012). Enhancement of bone regeneration through facile surface functionalization of solid freeform fabrication-based three-dimensional scaffolds using mussel adhesive proteins. *Acta Biomaterialia*.

[112] Kim HP, Ji YH, Rhee SC, Dhong ES, Park SH, Yoon ES (2012). Enhancement of bone regeneration using osteogenic-induced adipose-derived stem cells combined with demineralized bone matrix in a rat critically-sized calvarial defect model. *Current Stem Cell Research and Therapy*.

[113] Kim JY, Jin GZ, Park IS (2010). Evaluation of solid free-form fabrication-based scaffolds seeded with osteoblasts and human umbilical vein endothelial cells for use in vivo osteogenesis. *Tissue Engineering A*.

[114] Ko E, Yang K, Shin J, Cho SW (2013). Polydopamine-assisted osteoinductive Peptide immobilization of polymer scaffolds for enhanced bone regeneration by human adipose-derived stem cells. *Biomacromolecules*.

[115] Levi B, James AW, Nelson ER (2011). Studies in adipose-derived stromal cells: migration and participation in repair of cranial injury after systemic injection. *Plastic and Reconstructive Surgery*.

[116] Levi B, James AW, Nelson ER (2011). Acute skeletal injury is necessary for human adipose-derived stromal cell-mediated calvarial regeneration. *Plastic and Reconstructive Surgery*.

[117] Levi B, Nelson ER, Li S (2011). Dura mater stimulates human adipose-derived stromal cells to undergo bone formation in mouse calvarial defects. *Stem Cells*.

[118] Levi B, James AW, Nelson ER (2011). Human adipose-derived stromal cells stimulate autogenous skeletal repair via paracrine hedgehog signaling with calvarial osteoblasts. *Stem Cells and Development*.

[119] Levi B, Hyun JS, Nelson ER (2011). Nonintegrating knockdown and customized scaffold design enhances human adipose-derived stem cells in skeletal repair. *Stem Cells*.

[120] Lin CY, Chang YH, Li KC (2013). The use of ASCs engineered to express BMP2 or TGF-beta3 within scaffold constructs to promote calvarial bone repair. *Biomaterials*.

[121] Liu G, Zhang Y, Liu B, Sun J, Li W, Cui : L (2013). Bone regeneration in a canine cranial model using allogeneic adipose derived stem cells and coral scaffold. *Biomaterials*.

[122] Lo DD, Hyun JS, Chung MT (2012). Repair of a critical-sized calvarial defect model using adipose-derived stromal cells harvested from lipoaspirate. *Journal of Visualized Experiments*.

[123] Rhee SC, Ji YH, Gharibjanian NA, Dhong ES, Park SH, Yoon ES (2011). In vivo evaluation of mixtures of uncultured freshly isolated adipose-derived stem cells and demineralized bone matrix for bone regeneration in a rat critically sized calvarial defect model. *Stem Cells and Development*.

[124] Smith DM, Cooper GM, Afifi AM (2011). Regenerative surgery in cranioplasty revisited: the role of adipose-derived stem cells and BMP-2. *Plastic and Reconstructive Surgery*.

[125] Yoon E, Dhar S, Chun DE, Gharibjanian NA, Evans GRD (2007). In vivo osteogenic potential of human adipose-derived stem cells/poly lactide-co-glycolic acid constructs for bone regeneration in a rat critical-sized calvarial defect model. *Tissue Engineering*.

[126] Jo CH, Yoon PW, Kim H, Kang KS, Yoon KS (2013). Comparative evaluation of in vivo osteogenic differentiation of fetal and adult mesenchymal stem cell in rat critical-sized femoral defect model. *Cell and Tissue Research*.

[127] Stockmann P, Park J, von Wilmowsky C (2012). Guided bone regeneration in pig calvarial bone defects using autologous mesenchymal stem/progenitor cells—a comparison of different tissue sources. *Journal of Cranio-Maxillofacial Surgery*.

[128] Choi JW, Park EJ, Shin HS, Shin IS, Ra JC, Koh KS (2012). In vivo differentiation of undifferentiated human adipose tissue-derived mesenchymal stem cells in critical-sized calvarial bone defects. *Annals of Plastic Surgery*.

[129] Chou YF, Zuk PA, Chang TL, Benhaim P, Wu BM (2011). Adipose-derived stem cells and BMP2. Part 1: BMP2-treated adipose-derived stem cells do not improve repair of segmental femoral defects. *Connective Tissue Research*.

[130] Li X, Liu H, Niu X (2012). The use of carbon nanotubes to induce osteogenic differentiation of human adipose-derived MSCs in vitro and ectopic bone formation in vivo. *Biomaterials*.

[131] Man Y, Wang P, Guo Y (2012). Angiogenic and osteogenic potential of platelet-rich plasma and adipose-derived stem cell laden alginate microspheres.. *Biomaterials*.

[132] Schubert T, Xhema D, Vériter S (2011). The enhanced performance of bone allografts using osteogenic-differentiated adipose-derived mesenchymal stem cells. *Biomaterials*.

[133] Shen FH, Werner BC, Liang H (2013). Implications of adipose-derived stromal cells in a 3D culture system for osteogenic differentiation: an in vitro and in vivo investigation. *The Spine Journal*.

[134] Qing W, Guang-Xing C, Lin G, Liu Y (2012). The osteogenic study of tissue engineering bone with BMP2 and BMP7 gene-modified rat adipose-derived stem cell. *Journal of Biomedicine and Biotechnology*.

[135] Cao Z, Hou S, Sun D, Wang X, Tang J (2012). Osteochondral regeneration by a bilayered construct in a cell-free or cell-based approach. *Biotechnology Letters*.

[136] Fernandez FB, Shenoy S, Suresh Babu S, Varma HK, John A (2012). Short-term studies using ceramic scaffolds in lapine model for osteochondral defect amelioration. *Biomedical Materials*.

[137] Hao W, Dong J, Jiang M, Wu J, Cui F, Zhou D (2010). Enhanced bone formation in large segmental radial defects by combining adipose-derived stem cells expressing bone morphogenetic protein 2 with nHA/RHLC/PLA scaffold. *International Orthopaedics*.

[138] Kang BJ, Ryu HH, Park SS (2012). Comparing the osteogenic potential of canine mesenchymal stem cells derived from adipose tissues, bone marrow, umbilical cord blood, and Wharton's jelly for treating bone defects. *Journal of Veterinary Science*.

[139] Keibl C, Fügl A, Zanoni G (2011). Human adipose derived stem cells reduce callus volume upon BMP-2 administration in bone regeneration. *Injury*.

[140] Lee SW, Padmanabhan P, Ray P (2009). Stem cell-mediated accelerated bone healing observed with in vivo molecular and small animal imaging technologies in a model of skeletal injury. *Journal of Orthopaedic Research*.

[141] Li J, Zhao Q, Wang E, Zhang C, Wang G, Yuan Q (2012). Transplantation of Cbfa1-overexpressing adipose stem cells together with vascularized periosteal flaps repair segmental bone defects. *Journal of Surgical Research*.

[142] Schubert T, Poilvache H, Galli C, Gianello P (2013). Dufrane D Galactosyl-knock-out engineered pig as a xenogenic donor source of adipose MSCs for bone regeneration. *Biomaterials*.

[143] Sheyn D, Pelled G, Zilberman Y (2008). Nonvirally engineered porcine adipose tissue-derived stem cells: use in posterior spinal fusion. *Stem Cells*.

[144] Sheyn D, Kallai I, Tawackoli W (2011). Gene-modified adult stem cells regenerate vertebral bone defect in a rat model. *Molecular Pharmaceutics*.

[145] Shoji T, Ii M, Mifune Y (2010). Local transplantation of human multipotent adipose-derived stem cells accelerates fracture healing via enhanced osteogenesis and angiogenesis. *Laboratory Investigation*.

[146] Parrilla C, Saulnier N, Bernardini C (2011). Undifferentiated human adipose tissue-derived stromal cells induce mandibular bone healing in rats. *Archives of Otolaryngology*.

[147] Wilson SM, Goldwasser MS, Clark SG (2012). Adipose-derived mesenchymal stem cells enhance healing of mandibular defects in the ramus of swine. *Journal of Oral and Maxillofacial Surgery*.

[148] Gimble JM, Guilak F, Bunnell BA (2010). Clinical and preclinical translation of cell-based therapies using adipose tissue-derived cells. *Stem Cell Research and Therapy*.

[149] Sandor GK, Tuovinen VJ, Wolff J (2013). Adipose stem cell tissue-engineered construct used to treat large anterior mandibular defect: a case report and review of the clinical application of good manufacturing practice-level adipose stem cells for bone regeneration. *Journal of Oral and Maxillofacial Surgery*.

[150] Abbott A (2013). Stem-cell ruling riles researchers. *Nature*.

[151] Casteilla L, Planat-Benard V, Laharrague P, Cousin B (2011). Adipose-derived stromal cells: their identity and uses in clinical trials, an update. *World Journal of Stem Cells*.

[152] Thesleff T, Lehtimäki K, Niskakangas T (2011). Cranioplasty with adipose-derived stem cells and biomaterial: a novel method for cranial reconstruction. *Neurosurgery*.

[153] Lendeckel S, Jödicke A, Christophis P (2004). Autologous stem cells (adipose) and fibrin glue used to treat widespread traumatic calvarial defects: case report. *Journal of Cranio-Maxillofacial Surgery*.

[154] Mesimäki K, Lindroos B, Törnwall J (2009). Novel maxillary reconstruction with ectopic bone formation by GMP adipose stem cells. *International Journal of Oral and Maxillofacial Surgery*.

[155] Bellotti C, Stanco D, Ragazzini S (2013). Analysis of the karyotype of expanded human adipose-derived stem cells for bone reconstruction of the maxillo-facial region. *International Journal of Immunopathology and Pharmacology*.

[156] Bianchi F, Maioli M, Leonardi E (2013). A new non-enzymatic method and device to obtain a fat tissue derivative highly enriched in pericyte-like elements by mild mechanical forces from human lipoaspirate. *Cell Transplant*.

